# Supplementary material for: Novel Apoptosis-Inducing Agents for the Treatment of Cancer, a New Arsenal in the Toolbox
Source: Cancers (Basel). 2019 Jul 31;11(8):1087. doi: 10.3390/cancers11081087 (PMC6721450; doi:10.3390/cancers11081087)
Supplement: Supplementary file 1 [file cancers-11-01087-s001.pdf]

Supplementary Materials

Novel Apoptosis-Inducing Agents for the Treatment of Cancer, a New Arsenal in the Toolbox

Bora Lim, Yoshimi Greer, Stanley Lipkowitz and Naoko Takebe

Table S1. First generation death receptor targeted therapeutics and the status of clinical trials.

Agents	Molecule Type	Target	Half Life	NCT# /Trial Phase	Current Status	Combinatorial Agents	Target Disease
Dulanermin (rhTRAIL)	recombinant human TRAIL	DR4/5	mean terminal phase half-life ranging from 0.56 to 1.02 h in human [1]	NCT00873756 (Phase 1), completed	discontinued	FOLFOX + Bevacizumab	advanced, recurrent, metastatic colorectal cancer
				Phase 1		single agent	advanced or metastatic solid tumors or non-Hodgkin's Lymphoma (NHL) without leukemic phase
				NCT00508625 (phase2), completed		treated with chemotherapy (paclitaxel, carboplatin) +/− bevacizumab	advanced non-small cell lung cancer
				NCT00400764 (Phase 1/2), terminated		rituximab	follicular and other low grade, CD20+, non-Hodgkin's lymphomas
Mapatumumab (HGS-ETR2)	fully human MAb	DR4	mean initial phase elimination half-life (t1/2,α) of 0.7 to 1.4 days, mean terminal phase elimination half-life (t1/2,β) ranges from 14.2 to 28.4 days in human [2]	Phase 1		single agent	advanced solid malignancies
				Phase 1		single agent	advanced solid malignancies
				Phase 1		gemcitabine + cisplatin	advanced solid malignancies
				NCT00092924 (phase2), completed		single agent	advanced non-small-cell lung cancer
				NCT00094848 (phase 1b/2), completed		single agent	relapsed or refractory non-Hodgkin's Lymphoma (NHL)
				NCT00583830 (phase2) completed		paclitaxel + carboplatin	advanced non-small-cell lung cancer
				phase2		single agent	refractory colorectal cancer

				NCT01088347 (phase 1/2), completed		cisplatin + radiation therapy	cervical cancer
				NCT01258608 (phase2), completed		sorafenib	hepatocellular carcinoma
Drozitumab (Apomab/ PRO95780)	fully human MAb	DR5	mean terminal half-life was 8.3 days in human [3]	Phase 1	discontinued	single agent	advanced malignancies
				NCT00480831 (phase 2), completed		paclitaxel + carboplatin + bevacizumab	previously untreated, advanced-stage non-small cell lung cancer
				NCT00543712 (phase 2), terminated		single agent	chondrosarcoma, non-small cell lung cancer
				NCT00497497 (phase1, completed)		cetuximab and irinotecan chemotherapy or the FOLFIRI regimen with bevacizumab	previously treated metastatic colorectal cancer
Conatumumab (AMG-655)	fully human MAb	DR5	mean terminal half-life of 13 to 19 days in human [4]	phase1	discontinued	single agent	advanced solid malignancies
				NCT01017822 (phase 1/2), withdrawn		gemcitabine hydrochloride, capecitabine, and radiation therapy	locally advanced pancreatic cancer
Lexatumumab (HGS-ETR2)	fully human MAb	DR5	mean terminal half-life of 15 days in human [5]	phase 1	discontinued	single agent	advanced solid malignancies
				phase 1		single agent	advanced solid malignancies and lymphoma
				phase 1		gemcitabine, pemetrexed, doxorubicin or FOLFIRI	various solid tumors
				NCT00428272 (phase 1, terminated)		with or without interferon gamma	pediatric solid tumors
HGS-TR2J	fully human MAb	DR5	not reported	phase 1 (Canada, unspecified route)	discontinued (see adisinsight.springer.com)	unclear	solid tumors
Tigatuzumab (CS-1008, TRA-8)	chimeric humanized mouse IgG	DR5	mean terminal half-life of 6-10 days in human [6]	NCT00320827 (phase 1), completed	discontinued	single agent	breast cancer, colorectal cancer, hepatocellular carcinoma, lymphoma; non-small cell lung cancer, ovarian cancer; pancreatic cancer
				NCT00945191 (phase 2), completed		paclitaxel + carboplatin	ovarian cancer

				NCT01033240 (phase 2), completed		sorafenib	advanced liver cancer
				NCT00521404 (phase 2), completed		gemcitabine	untreated, unresectable pancreatic cancer
				NCT00991796 (phase2), completed		paclitaxel + carboplatin	metastatic or unserectable NSCLC
				NCT01307891 (phase2), completed		abraxane (albumin–bound paclitaxel)	triple negative breast cancer
LBY135	chimeric humanized mouse IgG	DR5	mean elimination half-life of LBY135 ± capecitabine at saturation of clearance (≥10 mg/kg) ranged between 146 h and 492 h [7]		discontinued	capecitabine	solid tumors

Table S2. MCL1 and CDK9 inhibitor to induce apoptosis.

Agents and Ongoing Trials	Target Disease	Phase of the Trial	Combinatorial Agents	Supportive pre-Clinical Data/Mechanism of Action	Serial NCT Number
			**MCL1 inhibitors**		
AMG 176 first in human trial in subjects with relapsed or refractory Multiple Myeloma and subjects with relapsed or refractory Acute Myeloid Leukemia	Multiple myeloma, acute myeloid leukemia	I	Single agent	AMG 176 showed efficacy in MCL1 dependent cancers, such as multiple myeloma and AML pre-clinical models [8]	NCT02675452
Study of AZD5991 in Relapsed or Refractory Hematologic Malignancies.	Hematological malignancies	Phase I	Single agent	Preclinical study to discover and test efficacy in pre-clinical cancer models [9]	NCT03218683
			**CDK9 inhibitors**		
Phase I dose escalation of BCY1143572 in subjects with Acute Leukemia	Acute leukemia	I	Single agent	The activity of CDK9 inhibitor in acute leukemia [10]	NCT02345382
Open label phase I dose escalation study with BAY-1143572 in patients with advanced cancer	Metastatic solid tumors	I	Single agent	First potent selective inhibitor of exclusively transcription-regulating PTEFb/CDK9 [11]	NCT01938638
Study to assess safety, tolerability, pharmacokinetics and antitumor activity of AZD4573 in relapsed/ refractory hematological malignancies	Hematological malignancies	I	Single agents	Pre-clinical study to discover and test efficacy in pre-clinical cancer models [12]	NCT03263637

Table S3. IAP inhibitors that are currently studied in the clinic.

Agents and Ongoing Trials	Target Disease	Phase of the Trial	Combinatorial Agents	Supportive Pre-Clinical Data/Mechanism of Action	Serial NCT Number
**Non Smac mimetics IAP inhibitor**					
Phase I-2 study of ASTX660 in subjects with advanced solid tumors and lymphomas	Solid cancers and lymphomas	I/II	Single agent	Dual inhibitor of cIAP and XIAP	NCT02503423
**Birinapant**					
Birinapant and Carboplatin in treating patients with recurrent High Grade Ovarian, Fallopian Tube, or Primary Peritoneal Cancer	Gynecological cancers	I/II	Carboplatin	Birinapant potentiates the efficacy of carboplatin in platinum resistant IAP high solid cancers [13]	NCT02756130
Dose-escalation Study of Birinapant and Pembrolizumab in Solid Tumors	Metastatic solid tumors	Ib/II	Pembrolizumab	Smac mimetics inducing synergy with checkpoint inhibitor in GBM and other cancers [14]	NCT02587962
Study of birinapant in combination with conatumumab in subjects with relapsed ovarian cancer	Ovarian cancer	Ib	Conatumumab (AMG-655, anti-DR5 antibody)	TRAIL agonists synergistically induce cell apoptosis with Smac mimetics [15]	NCT01940172
Birinapant with 5-azacitidine (5-AZA) in myelodysplastic syndrome (MDS) subjects with who are naïve, have relapsed or are refractory to 5-AZA	MDS	Ib/II	5-AZA	Demethylating agent synergistically induce apoptosis in leukemic cells [16]	NCT01828346
Birinapant for advanced ovarian, fallopian tube and peritoneal cancer	Ovarian, fallopian tube and peritoneal cancer	I	Single agent	Smac mimetics induce efficacy in platinum resistant gynecological malignancies	NCT01681368
A phase I–II open label non-randomized study using TL32711 for patients with AML, MDS and ALL	AML, ALL, MDS	I/II	Single agent	Smac mimetics induce efficacy in therapy resistant hematological malignancies	NCT01486784
**LCL161**					
Study of single agent CJM112, and PDR001 in combination with LCL161 or CJM112 patients with Multiple Myeloma	Multiple myeloma	Ib	CJM112 (anti-IL17A monoclonal antibody), PDR001 (anti-PD1 antibody)	IAP and anti-PD1, and anti-IL17 and anti-PD1 synergistically modulate immune response in therapy resistant cancers and other inflammatory conditions [17]	NCT03111992
LCL161 plus topotecan for patients with relapsed/ refractory SCLC and select Gynecologic malignancies	SCLC, gynecological malignancies	Ib with dose expansion	Topotecan	Smac mimetics induce synergy with topotecan induced DNA damage-based cancer death	NCT02649673
A study of PDR001 in combination with LCL161, everolimus or panobinostat	Colorectal cancer, NSCLC, TNBC	Ib	PDR001 (anti-PD1 antibody)	HDAC inhibitor induces synergy with Smac mimetics in Smac mimetics resistance in AML and mesothelioma [18,19]	NCT02890069
SMAC mimetics LCL161 alone with cyclophosphamide in treating patients with relapsed/ refractory Multiple Myeloma	Multiple myeloma	Ib/II	cyclophosphamide	Smac mimetics overcomes the resistance to the chemotherapy in multiple myeloma [20]	NCT01955434
Phase I trial of LCL161 and gemcitabine plus nab-paclitaxel in Metastatic Prostate Cancer	Metastatic prostate cancer	Ib	Gemcitabine and nab-paclitaxel	Induction of effective death in prostate cancer by modulating IAP and NF-kB [21]	NCT01934634
A randomized, phase II neoadjuvant study of weekly paclitaxel with or without LCL161 in patients with TNBC	TNBC	II	Paclitaxel	Chemotherapy and birinapant showed synergy in TNBC pre-clinical model [22]	NCT01617668

Reference

1. Herbst, R.S.; Eckhardt, S.G.; Kurzrock, R.; Ebbinghaus, S.; O'Dwyer, P.J.; Gordon, M.S.; Novotny, W.; Goldwasser, M.A.; Tohnya, T.M.; Lum, B.L.; et al. Phase i dose-escalation study of recombinant human apo2l/trail, a dual proapoptotic receptor agonist, in patients with advanced cancer. *J. Clin. Oncol.* **2010**, *28*, 2839–2846.
2. Hotte, S.J.; Hirte, H.W.; Chen, E.X.; Siu, L.L.; Le, L.H.; Corey, A.; Iacobucci, A.; MacLean, M.; Lo, L.; Fox, N.L.; et al. A phase 1 study of mapatumumab (fully human monoclonal antibody to trail-r1) in patients with advanced solid malignancies. *Clin. Cancer Res.* **2008**, *14*, 3450–3455.
3. Merchant, M.S.; Geller, J.I.; Baird, K.; Chou, A.J.; Galli, S.; Charles, A.; Amaoko, M.; Rhee, E.H.; Price, A.; Wexler, L.H.; et al. Phase i trial and pharmacokinetic study of lexatumumab in pediatric patients with solid tumors. *J. Clin. Oncol.* **2012**, *30*, 4141–4147.
4. Herbst, R.S.; Kurzrock, R.; Hong, D.S.; Valdivieso, M.; Hsu, C.P.; Goyal, L.; Juan, G.; Hwang, Y.C.; Wong, S.; Hill, J.S.; et al. A first-in-human study of conatumumab in adult patients with advanced solid tumors. *Clin. Cancer Res.* **2010**, *16*, 5883–5891.
5. Wakelee, H.A.; Patnaik, A.; Sikic, B.I.; Mita, M.; Fox, N.L.; Miceli, R.; Ullrich, S.J.; Fisher, G.A.; Tolcher, A.W. Phase i and pharmacokinetic study of lexatumumab (hgs-etr2) given every 2 weeks in patients with advanced solid tumors. *Ann. Oncol.* **2010**, *21*, 376–381.
6. Forero-Torres, A.; Shah, J.; Wood, T.; Posey, J.; Carlisle, R.; Copigneaux, C.; Luo, F.R.; Wojtowicz-Praga, S.; Percent, I.; Saleh, M. Phase i trial of weekly tigatuzumab, an agonistic humanized monoclonal antibody targeting death receptor 5 (DR5). *Cancer Biother. Radiopharm.* **2010**, *25*, 13–19.
7. Sharma, S.; de Vries, E.G.; Infante, J.R.; Oldenhuis, C.N.; Gietema, J.A.; Yang, L.; Bilic, S.; Parker, K.; Goldbrunner, M.; Scott, J.W.; et al. Safety, pharmacokinetics, and pharmacodynamics of the dr5 antibody lby135 alone and in combination with capecitabine in patients with advanced solid tumors. *Invest. New Drugs* **2014**, *32*, 135–144.
8. Caenepeel, S.R.; Belmontes, B.; Sun, J.; Coxon, A.; Moody, G.; Hughes, P.E. Abstract 2027: Preclinical evaluation of AMG 176, a novel, potent and selective Mcl-1 inhibitor with robust anti-tumor activity in Mcl-1 dependent cancer models. *Cancer Res.* **2017**, *77*, Supplement 2027, doi:10.1158/1538-7445.AM2017-2027.
9. Hird, A.W.; Secrist, J.P.; Adam, A.; Belmonte, M.A.; Gangl, E.; Gibbons, E.F.; Hargreaves, D.; Johannes, J.W.; Kazmirski, S.L.; Kettle, J.G.; et al. Abstract DDT01-02: AZD5991: A potent and selective macrocyclic inhibitor of Mcl-1 for treatment of hematologic cancers. *Cancer Res.* **2017**, *77*, Supplement DDT01-02, doi:10.1158/1538-7445.AM2017-DDT01-02.
10. Narita, T.; Ishida, T.; Ito, A.; Masaki, A.; Kinoshita, S.; Suzuki, S.; Takino, H.; Yoshida, T.; Ri, M.; Kusumoto, S.; et al. Cyclin-dependent kinase 9 is a novel specific molecular target in adult T-cell leukemia/lymphoma. *Blood* **2017**, *130*, 1114–1124, doi:10.1182/blood-2016-09-741983.
11. Lucking, U.; Scholz, A.; Lienau, P.; Siemeister, G.; Kosemund, D.; Bohlmann, R.; Briem, H.; Terebesi, I.; Meyer, K.; Prelle, K.; et al. Identification of Atuveciclib (BAY 1143572), the First Highly Selective, Clinical PTEFb/CDK9 Inhibitor for the Treatment of Cancer. *Chem. Med. Chem.* **2017**, *12*, 1776–1793, doi:10.1002/cmdc.201700447.
12. Barlaam, B.; De Savi, C.; Drew, L.; Ferguson, A.D.; Ferguson, D.; Gu, C.; Hawkins, J.; Hird, A.W.; Lamb, M.L.; O'Connell, N.; et al. Abstract 1650: Discovery of AZD4573, a potent and selective inhibitor of CDK9 that enables transient target engagement for the treatment of hematologic malignancies. **2018**, 10.1158/1538-7445.AM2018-1650, doi:10.1158/1538-7445.AM2018-1650.
13. La, V.; Fujikawa, R.; Janzen, D.M.; Nunez, M.; Bainvoll, L.; Hwang, L.; Faull, K.; Lawson, G.; Memarzadeh, S. Birinapant sensitizes platinum-resistant carcinomas with high levels of cIAP to carboplatin therapy. *NPJ Precis. Oncol.* **2017**, *1*, doi:10.1038/s41698-017-0008-z.
14. Beug, S.T.; Beauregard, C.E.; Healy, C.; Sanda, T.; St-Jean, M.; Chabot, J.; Walker, D.E.; Mohan, A.; Earl, N.; Lun, X.; et al. Smac mimetics synergize with immune checkpoint inhibitors to promote tumour immunity against glioblastoma. *Nat. Commun.* **2017**, *8*, doi:10.1038/ncomms14278.
15. Raulf, N.; El-Attar, R.; Kulms, D.; Lecis, D.; Delia, D.; Walczak, H.; Papenfuss, K.; Odell, E.; Tavassoli, M. Differential response of head and neck cancer cell lines to TRAIL or Smac mimetics is associated with the cellular levels and activity of caspase-8 and caspase-10. *Br. J. Cancer* **2014**, *111*, 1955–1964, doi:10.1038/bjc.2014.521.

16. Steinhart, L.; Belz, K.; Fulda, S. Smac mimetic and demethylating agents synergistically trigger cell death in acute myeloid leukemia cells and overcome apoptosis resistance by inducing necroptosis. *Cell Death Dis.* **2013**, *4*, e802, doi:10.1038/cddis.2013.320.
17. Wang, C.Q.F.; Akalu, Y.T.; Suarez-Farinas, M.; Gonzalez, J.; Mitsui, H.; Lowes, M.A.; Orlow, S.J.; Manga, P.; Krueger, J.G. IL-17 and TNF synergistically modulate cytokine expression while suppressing melanogenesis: potential relevance to psoriasis. *J. Invest. Dermatol.* **2013**, *133*, 2741–2752, doi:10.1038/jid.2013.237.
18. Steinwascher, S.; Nugues, A.L.; Schoeneberger, H.; Fulda, S. Identification of a novel synergistic induction of cell death by Smac mimetic and HDAC inhibitors in acute myeloid leukemia cells. *Cancer Lett.* **2015**, *366*, 32–43, doi:10.1016/j.canlet.2015.05.020.
19. Crawford, N.; Stasik, I.; Holohan, C.; Majkut, J.; McGrath, M.; Johnston, P.G.; Chessari, G.; Ward, G.A.; Waugh, D.J.; Fennell, D.A.; et al. SAHA overcomes FLIP-mediated inhibition of SMAC mimetic-induced apoptosis in mesothelioma. *Cell Death Dis.* **2013**, *4*, e733, doi:10.1038/cddis.2013.258.
20. Ramakrishnan, V.; Gomez, M.; Prasad, V.; Kimlinger, T.; Painuly, U.; Mukhopadhyay, B.; Haug, J.; Bi, L.; Rajkumar, S.V.; Kumar, S. Smac mimetic LCL161 overcomes protective ER stress induced by obatoclax, synergistically causing cell death in multiple myeloma. *Oncotarget* **2016**, *7*, 56253–56265, doi:10.18632/oncotarget.11028.
21. Dai, Y.; Liu, M.; Tang, W.; Li, Y.; Lian, J.; Lawrence, T.S.; Xu, L. A Smac-mimetic sensitizes prostate cancer cells to TRAIL-induced apoptosis via modulating both IAPs and NF-kappaB. *BMC Cancer* **2009**, *9*, 392, doi:10.1186/1471-2407-9-392.
22. Min, D.J.; He, S.; Green, J.E. Birinapant (TL32711) Improves Responses to GEM/AZD7762 Combination Therapy in Triple-negative Breast Cancer Cell Lines. *Anticancer Res.* **2016**, *36*, 2649–2657.

© 2019 by the authors. Licensee MDPI, Basel, Switzerland. This article is an open access article distributed under the terms and conditions of the Creative Commons Attribution (CC BY) license (http://creativecommons.org/licenses/by/4.0/).
